# Posterior Mediastinal Hematoma after a Fall from Standing Height: A Case Report

**DOI:** 10.1155/2012/672370

**Published:** 2012-03-05

**Authors:** J. M. Josse, A. Ameer, S. Alzaid, A. Arrowaili, M. Ben-Ely, D. L. Decklebaum, P. Fata, T. Razek, K. Khwaja

**Affiliations:** ^1^Ross University School of Medicine, 630 US Highway 1, North Brunswick, NJ 08902, USA; ^2^McGill University Medical Center, The Montreal General Hospital, 1650 Cedar Avenue, Montreal, QC, Canada H3G 1A4; ^3^Trauma Program, Trauma Surgery and Critical Care Medicine, McGill University Health Centre, 1650 Cedar Avenue, L9.411, Montreal, QC, Canada H3G 1A4

## Abstract

Posterior Mediastinal Hematomas (PMHs) secondary to a fall from standing height are uncommon, with only one previous case reported in the literature. We describe a case of a 78-year-old male with multiple medical comorbidities, who was transferred to Montreal General Hospital (MGH) with a posterior mediastinal hematoma (PMH) after sustaining a fall from standing height. On initial assessment, the patient was hemodynamically stable and complained of mild chest pain, dyspnea, fatigue, and diaphoresis. The patient's airway was secured via endotracheal intubation fearing impending respiratory compromise secondary to an enlarging PMH. The patient was admitted to ICU where over the next 3 days he was managed conservatively via careful monitoring of his hemodynamic and hematologic indices. Repeat CT scanning indicated reduction in size of the PMH. The patient was discharged on hospital day eight. This case describes the assessment, evaluation, and conservative management of PMH in a complicated patient receiving prior anticoagulation. A review of the literature regarding the epidemiology of PMH and the management of both unstable and stable PMHs is also presented.

## 1. Introduction

The posterior mediastinum is an anatomical region bordered superiorly via the transverse thoracic plane, created by the angle of the sternum anteriorly and the fourth and fifth thoracic vertebrae posteriorly [1]. The inferior margin of the posterior mediastinum is bound by the thoracic surface of the diaphragm. The pericardium and vertebral bodies demarcate the anterior and posterior boarders of the posterior mediastinum, respectively [1]. Contained within the posterior mediastinum are the thoracic aorta, thoracic duct, lymphatic trunks and posterior mediastinal lymph nodes, azygos and hemiazygos veins, and esophagus and esophageal nerve plexus [1]. The mediastinum is a highly mobile region and consists of mainly hollow visceral organs which allow for considerable movement in conjunction with respiration, deglutition, or muscular contraction [1]. As a consequence of this mobility, the potential for the creation of a false cavity for the collection of fluids such as air, blood, or lymph can occur as a result of trauma.

A review of the literature from 1980 to present identified 46 cases of posterior mediastinal hematoma (PMH), with 9 cases secondary to blunt trauma and one case secondary to a fall from a standing height. Injury etiologies giving rise to PMH are largely subdivided into traumatic and nontraumatic causes. Traumatic PMH can be further subdivided into blunt, penetrating, and iatrogenic causes [2]. With respect to blunt trauma and in the absence of aortic injury, venous injury to structures such as the azygos, hemiazygos, vena cava, and small venous tributaries of the internal mammary, brachiocephalic and inferior thyroidal veins can result in the development of hematoma [2–5]. Previous literature has sited that these smaller vessels are typically anchored indirectly to the chest wall and are therefore susceptible to shearing forces as a result of trauma [2]. In particular, mediastinal hematoma is common among patients with vertebral fractures. Literature cited by Earls et al. (1997) stated that approximately 66% of patients with vertebral body fractures to C6-T8 can show signs of mediastinal hematoma (e.g., mediastinal widening) on the anteroposterior (AP) chest radiograph. However, a widened mediastinum on AP chest radiograph is a nonspecific finding [6].

## 2. Case Presentation

 A 78-year-old male with a past medical history of hypertension, cerebral vascular accident with residual right-sided deficit, atrial fibrillation on Coumadin therapy, and Guillain-Barré syndrome presented to his nearest hospital after sustaining a fall from standing height. The patient stated that while walking to his bathroom, he lost his balance, fell and struck his chest on his toilet. Prior to the fall, the patient denied any history of chest pain, shortness of breath, dizziness, syncope, paresthesias, extremity weakness, or vertigo.

 During the course of the initial diagnostic work-up at a local medical center, the patient refused several evaluations and requested to be discharged home, citing a resolution of symptoms. Approximately two days later, the patient represented this facility with symptoms of mild chest pain, diaphoresis, dysphagia, dysphonia, and fatigue. Vital signs at the time of presentation were stable and Computed Tomographic (CT) scan of the chest showed a posterior mediastinal hematoma measuring 4.6 cm × 5.5 cm × 2.2 cm, with corresponding mass effect compressing the Trachea. No acute thoracic aortic injury was identified on CT scan. The patient received Human Prothrombin Complex (“Octaplex”—Octapharma Pharazeutika Produktionsges) and Vitamin K and was transferred to the Montreal General Hospital (MGH), where he was admitted to the Trauma Surgery service for evaluation. On presentation to MGH, the patient's vital signs were as follows: blood pressure 155/83, heart rate 68 beats/min (regular), oxygen saturation of 95% on 2.5 L of oxygen via nasal cannula, and a respiration rate of 18 breaths/min. Additional pertinent findings included an INR of 3.4 and a Glasgow Coma Scale of 15/15.

**Figure 1:**
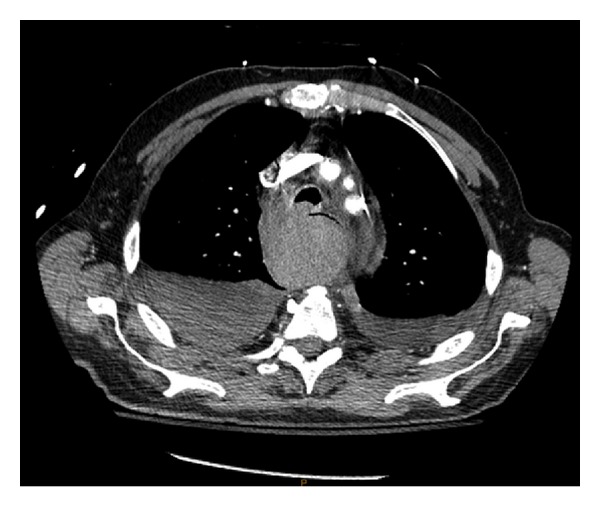
Posterior Mediastinal Hematoma measuring 5.9 cm × 5.8 cm × 21 cm, identified via CT scan upon patient presentation to MGH.

**Figure 2:**
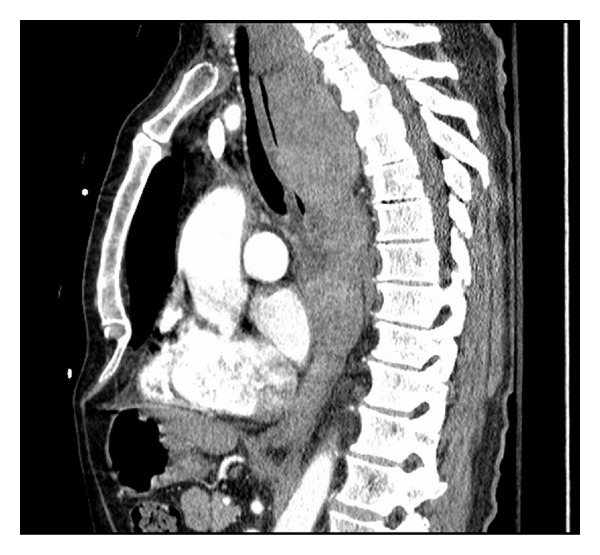
Sagittal view of a Posterior Mediastinal Hematoma measuring 5.9 cm × 5.8 cm × 21 cm, identified via CT scan upon patient presentation to MGH.

 Repeat CT scan at MGH confirmed the presence of a posterior mediastinal hematoma with bilateral hemopneumothorax, in addition to a fracture of the inferior right facet joint of the C7 vertebrae (not shown) (Figures 1 and 2). The patient was intubated in the ER due to concern regarding the possibility airway compromise, secondary to an expanding hematoma. The patient was then transferred to the ICU where initial management included a complete blood count (continued in series), c-spine immobilization with a rigid cervical collar and cessation of all anticoagulation medication, with concurrent rate control for the patient's preexisting atrial fibrillation.

**Figure 3:**
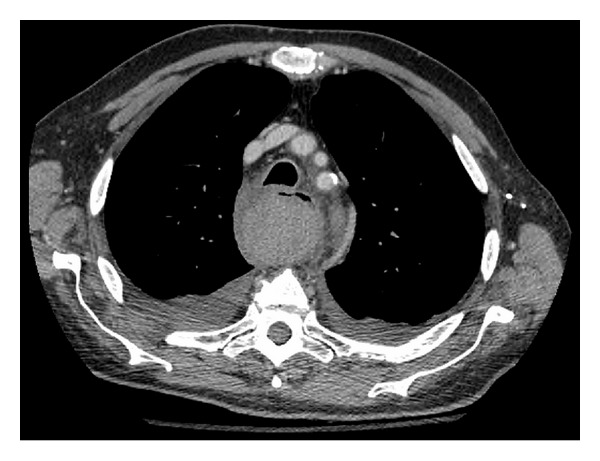
Repeat CT scan of a Posterior Mediastinal Hematoma measuring 4.6 cm × 4.6 cm × 18.5 cm, two days after patient presentation to MGH.

 On day one of admission, the patient's vital signs and hemoglobin levels remained stable. The patient was extubated without difficulty. Repeat CT scans of the chest indicated no enlargement of the hematoma or structural abnormalities of the Thoracic aorta (i.e., dissection, aneurysm). Consults to orthopedic surgery and cardiothoracic surgery were placed to assess the patient's cervical fracture and hematoma. It was determined that since both injuries were stable, conservative management would be maintained. Daily chest X-rays were ordered to assess the resolution of the patient's hemothoraces. Evacuation of the hemothoraces via chest tube drainage was considered but deferred, as follow-up CT scans on Hospital days (HDs) two and four demonstrated a decrease in the size of both the hematoma and hemothoraces (Figure 3). The patient was transferred from the ICU to a surgical floor on HD two. Physical examination of the patient on HD four demonstrated stable vital signs, resolution of chest pain, and no reports of dysphagia, dyspnea, or dysphonia.

Subsequent consultations to the hematology service were made with respect to resumption of the patient's Coumadin therapy. The hematology service recommended against restarting the patient's Coumadin therapy, instead electing manage his atrial fibrillation via rate control with Metoprolol. The patient was discharged home on HD eight with instructions to return to the nearest Emergency room if symptoms worsened. Follow-up CT scans were scheduled in two weeks to assess the resolution of his hematoma. The patient was referred for outpatient hematology follow-up to manage his anticoagulation status.

## 3. Discussion

 The most common presenting symptoms among patients who are hemodynamically stable with a mediastinal hematoma are shortness of breath and chest pain [5]. Additional presenting symptoms may include (but are not limited to) respiratory distress, hypotension, tachycardia, chest wall ecchymosis [5, 7], and dysphagia possibly secondary to compression of the esophagus by an expanding hematoma.

Patients presenting with a traumatic aortic injury (TAI), versus a venous mediastinal bleed as a result of trauma, can be differentiated by several clinical and radiological factors. Only a small proportion of TAI patients survive to receive medical attention. The mortality rates of an untreated TAI see 30% of patients dying within 6 hours, 40–50% dying within 24 hours, and 90% dying within 4 months [2]. In patients with a TAI who survive to present to hospital, these patients can complain of severe chest pain with radiation to the back or display signs of shock and hypotension. Chest X-ray is the standard initial screening test to assess patients with thoracic injuries. As described by Rojas et al. (2009), the radiological signs indicating a possible arterial mediastinal injury include widening of the superior mediastinum (>8 cm) at the level of the aortic arch The presence of a blush or shadow at the left lung apex (i.e., an “apical cap”), widening of the right paratracheal stripe (≥5 cm), and deviation of the nasogastric tube (if placed) to the right of the T4 spinous process.

 Current standard of care requires a computed tomography (CT) scan of the chest to assess for mediastinal injury. The advantages for CT scan can allow for a rapid and noninvasive evaluation of the mediastinum. Multidetector contrast enhanced CT has been cited to approach 100% sensitivity and demonstrates a similar negative predictive value with respect to the detection of traumatic aortic injury [2]. Disadvantages to CT scanning mainly encompass its inability to identify venous sources of hemorrhage. However, venous bleeds are fortunately likely to be moderate in size and self-contained [2]. An alternate modality for evaluating mediastinal widening with suspected vascular injury is transesophageal echocardiography (TEE). Though mainly employed to assess for traumatic disruption of the thoracic aorta, TEE can allow for evaluation of the innominate artery, left common carotid artery, intercostal arteries, and left subclavian artery [8].

 Treatment of posterior mediastinal hematomas centers around two broad categories: operative and conservative management. The former is typically employed in lieu of a significant mediastinal bleed (i.e., arterial) and compromised hemodynamic stability of the patient. Initial management of unstable patients include securing the airway via endotracheal intubation, and establishing peripheral access with large bore venous catherization or central line placement and resuscitation with fluids, blood transfusions, and reversal of coagulopathy, if required. Radiological assessment via CT scanning remains the gold standard in the acute setting. However, identification of the site of bleeding may be achieved through angiography and hemodynamic control can be accomplished via embolization. If unsuccessful, patients may require operative exploration via left-sided thoracotomy, with repair and evacuation of any blood within the mediastinum [9, 10]. However, a review of the literature indicated one case of successful evacuation of a PMH in a hemodynamically stable patient, via 3-port access thoracoscopy [11]. In the context of evolving dyspnea, dysphagia, or airway compromise, the application of minimally invasive thoracoscopic drainage or radiologically assisted drainage may be applicable, though further research is warranted given the relative rarity of such cases and the potential morbidity associated with these interventions.

Conservative management, as employed in this case, involved implementation of Advanced Trauma Life Support (ATLS) protocols on presentation with CT scanning to identify the site and degree of extravasation. Constant hemodynamic monitoring should be maintained in the intensive care and surgical step-down units. In stable patients, serial examination of the patient's hematologic indices can suggest a continued bleed, as manifested by persistent decreases in hemoglobin levels. Furthermore, repeat CT scans can assess the degree of resolution of the hematoma. Long-term sequelae resulting from PMH are rare within the literature. Patient's with a prior history of traumatic thoracic hemrhorage maybe predisposed towards the development of fibrosing mediastinitis [12]. This may lead to the progressive entrapment and compromise of the esophagus, airways, and low-pressure venous structures. In these cases, surgical repair of the obstruction is indicated [13].

## 4. Conclusion

 Falls from a standing height are a common occurrence among the elderly. A careful examination of injury mechanisms and patient presention will dictate whether the patient requires emergent invasive intervention, or if the patient can be managed conservativedly. This paper outlines the successful management of a PMH in a patient with prior anticoagulation and symptoms of an enlarging hematoma. With respect to this case, diligent monitoring of the patient's vital signs and hematologic indices, in addition to serial radiological examinations, were paramount. Determinations regarding the reinstitution of anticoagulation should be made with careful consideration of patient characteristics, in concert with consultation with the appropriate authorities.
